# Endoscopic assisted adenoidectomy versus conventional curettage adenoidectomy: a meta-analysis of randomized controlled trials

**DOI:** 10.1186/s40064-016-2072-1

**Published:** 2016-04-11

**Authors:** Liyun Yang, Yamin Shan, Shili Wang, Changping Cai, Hao Zhang

**Affiliations:** Department of Otolaryngology, Ruijin Hospital, School of Medicine, Shanghai Jiaotong University, 197, Ruijin 2nd Road, Shanghai, 200025 China

**Keywords:** Endoscopy, Curettage, Meta-analysis, Operative time, Blood loss, Complications

## Abstract

Adenoidectomy, surgical removal of hypertrophic adenoids, is a common operation in children worldwide. The purpose of this study was to compare the operative effectiveness, and included total operative time, blood loss and complications, between endoscopic assisted adenoidectomy and conventional curettage adenoidectomy. EMBASE, PubMed, Cochrane Library, and China National Knowledge Infrastructure and symposiums and review articles were used to choose relevant randomized controlled trials. A meta-analysis was performed to analyze the data for total operative time, blood loss and complications. Seven studies fit the inclusion criteria, and included 331 patients treated with endoscopic assisted adenoidectomy, and 251 patients treated with conventional curettage adenoidectomy. The meta-analysis demonstrated that compared with conventional curettage adenoidectomy, endoscopic assisted adenoidectomy had a shorter operative time (SMD −1.09; 95 % CI −1.29 to −0.90; p < 0.00001), less blood loss (MD −19.74; 95 % CI −22.75 to −16.73; p < 0.00001), and fewer complications (OR 0.15; 95 % CI 0.07–0.35; p < 0.0001). Endoscopic assisted adenoidectomy has advantages over conventional curettage adenoidectomy with regard to total operative time, blood loss and complications.

## Background

With of approximately 250,000 cases, adenoidectomy remains one of the surgical procedures most frequently performed by otolaryngologist in the United States (Spencer and Jones 2012). The indications for adenoidectomy include children who have recrudescent or chronic otitis media, chronic rhinosinusitis, nasopharyngeal obstruction causing sleep maladjustments and consecutive mouth breathing. The objective of adenoidectomy is to remove an infected or enlarged and obstructive adenoid.

Historically recommended instrumentation for performing adenoidectomy has varied from the surgeon’s fingernail, a steel nail, cutting or biting forceps, adenotomes and adenoid curettes (Jonas et al. 2007). The conventional curettage adenoidectomy was first described in 1885 (Thornval 1969), and since then it has been considered the most commonly used surgical technique for adenoidectomy (Costantini et al. 2008). Conventional curettage for removing adenoids uses the nasopharyngeal touch method to estimate the size of the adenoid and the relationship to the surrounding structure, in order to choose the suitable adenoidectomy curette to scrape the adenoid tissue in the rhinopharynx transorally behind the nose.

Over time, a considerable number of instruments have been employed to perform adenoidectomy, including an electronic molecular resonance tool, suction diathermy, a microdebrider, endoscopy and laser (Tarantino et al. 2004; Walker 2001; Sorin et al. 2004; Shin and Hartnick 2003; Ozkiris et al. 2013). At present, endoscopic assisted adenoidectomy involves general anesthesia, followed by the application of a microdebrider with irrigating blades of different angles (0°, 15°, 45° and 60°) or special a adenoid blade and a 0° 2.7-mm rigid telescope (4 mm for older children) to shave the adenoid (Somani et al. 2010).

Despite improvements in the surgery, the complications of adenoidectomy are often inevitable. Primary and secondary hemorrhages are the major complication for all patients undergoing adenoidectomy. Minor complication, including fever, soreness, dehydration, refractory emesis, and neck stiffness also are often seen postoperatively. In view of the large number of adenoidectomies performed, the otolaryngologist should pay close attention to the surgical method. The choice of the endoscopic assisted adenoidectomy versus conventional curettage adenoidectomy has been widely debated.

Conventional curettage adenoidectomy can be performed in any hospital, especially in those that do not possess advanced instruments. Moreover, the cost of conventional curettage adenoidectomy is very low, so the majority of patients can afford it, particularly poor families in developing countries. However the conventional curettage adenoidectomy for removing adenoids is a relatively ‘blind’ technique which risks nasopharyngeal injury and incomplete adenoid removal (Regmi et al. 2011). By measuring the volume of residual adenoid tissues, both Saxby and Chappel (2009) and Cannon et al. (1999) objectively proved that conventional curettage adenoidectomy misses a substantial amount of adenoid tissue. A prospective study involving endoscopic evaluation of cases operated by curette and microdebrider showed that conventional curettage adenoidectomy was less precise than endoscopic assisted adenoidectomy, especially in the choanal and tubaric regions (Owens et al. 2005). Compared with conventional curettage adenoidectomy, Havas and Lowinger (2002) also demonstrated that endoscopic assisted adenoidectomy had was superior to conventional curettage adenoidectomy for complete removal of adenoids in a shorter operative time. However, one study by Elnashar et al. (2014) stated that there was no difference in effectiveness between the two methods when under grade 3 adenoid enlargement on X-ray. However, Songu et al. (2010) stated that conventional adenoidectomy was better than endoscopic assisted adenoidectomy with regard to blood loss and complications.

Although many studies have compared endoscopic assisted adenoidectomy and conventional curettage adenoidectomy, there is as yet no agreement on which technique is superior. Therefore, we conducted a meta-analysis in an attempt to resolve this issue.

## Methods

This study was conducted according to the PRISMA (Preferred Reporting Items for Systematic Reviews) for statement (Moher et al. 2010).

### Literature search

EMBASE, PubMed, Cochrane Library (CL), and China National Knowledge Infrastructure databases, and symposiums and review articles were used to identify relevant randomized controlled trials from the day they initiated until October 2014, with no limit for language types. Search keywords were as follows: “adenoidectomy”, “endoscopic assisted adenoidectomy” “conventional curettage adenoidectomy”, “classical adenoid curette”, “power assisted adenoidectomy”, “adenoid hypertrophy surgery”, “cohort study”, “prospective study”, and “randomized control trial”. If there are no original data, we contacted authors directly to obtain it. If we can not get the original data from the authors, and the study is so important for the analysis, we can use a formula to calculate according to the available data (Hozo et al. 2005).

### Study selection

All studies compared endoscopic assisted adenoidectomy and conventional curettage adenoidectomy. Suitable studies are selected according to the following criteria (1) the study is reported at least one of the following outcomes: total operative time, blood loss or complications; (2)the study is obtained available data; (3) the study is irrespective of age, sex, weight and height. Reasons for study exclusion included the following (1) patients had systemic diseases; (2) the study is only reported as an abstract or with incorrect data; (3) the study is used a sample size <10; (4) the standard used to assess curative effect is not stated.

### Data extraction

Two researchers (L.Y. and Y.M.) independently selected the eligible studies, and then a table was designed to extract their characteristics. If there was any disagreement between the two researchers, we sent it to a third reviewer (Z.H.) to resolve. The salient characteristics included the first author’s name, the year published, basic demographics of participants, study method and outcomes (Table 1).

**Table 1:** The basic characteristics of eligible studies

Name year: Khalid A. Al-Mazrou 2009
Methods: RCT (double-blind)
Participants: 40 children (age ranged from 3 to 17 years, from 2002 to 2003) with symptoms and signs suggestive of snoring and/or obstructive sleep apnea and adenoid hypertrophy, any patient with recurrent adenoid enlargement, bleeding tendency, or with sever bilateral deviated nasal septum were excluded
Interventions: endoscopic powered adenoidectomy versus curettage adenoidectomy
Outcomes: the mean blood loss, operative time, operative or postoperative complications (postoperative follow up of all patients was from 3 to 24 months, median of 6 months)
Name year: Murat Songu, MD 2010
Methods: RCT (double blind)
Participants: 38 patients who underwent adenoidectomy alone or in combination (age ranged from 8 to 12 years old) study was performed from April 2008 to September 2009
Interventions: endoscopic assisted adenoidectomy versus curettage adenoidectomy
Outcomes: adenoidectomy/nasopharyngeal ratios, operative time, blood loss, symptom improvement
Name year: Özmen Öztürk · Şenol Polat 2012
Methods: RCT (no blind)
Participants: 53 patients (younger than 16 years, with the presence of nasal airway obstruction with sleep disordered breathing, otitis media with effusion or recurrent otitis media, and chronic or recurrent rhinosinusitis). Completed the study (the 6 months follow-up) between, the study performed from January 2004 to December 2010
Interventions: powered-assisted endoscopic adenoidectomy versus curettage adenoidectomy
Outcomes: VAS score, score improvement, the average ratio of choanal opening obstructed, the reduction of adenoid size
Name year: Paul Stanislaw 2000
Methods: RCT (unclear)
Participants: 90 patients (age from 1 to 13 years old) underwent power assisted adenoidectomy and 87 patients (age from 1 to 12 years old) underwent conventional curettage adenoidectomy
Interventions: power assisted adenoidectomy versus conventional curettage adenoidectomy
Outcomes: operative time, blood loss, completeness and depth of resection, injuries to surrounding structures, short and long term complication, surgeon satisfaction with the procedure and patients’ postoperative recovery period
Name year: Nicole Murray 2002
Methods: RCT (unclear)
Participants: 100 children underwent powered partial adenoidectomy and 40 children underwent conventional partial adenoidectomy, the study period from October 1997 to July 1998. All patients younger than 20 years old
Interventions: powered partial adenoidectomy versus conventional partial adenoidectomy
Outcomes: operative time (specific quantification of the time removal and hemostasis), blood loss, complications, adenoid size, plate length, submucus cleft stigmata
Name year: Zhang G. Y. 2013
Methods: RCT (single blind)
Participants: 50 patients (age from 1 to 18 years old) underwent endoscopic assisted adenoidectomy and 50 patients (age from 1 to 18 years old) underwent conventional curettage adenoidectomy (period from January 2008 to December 2011), adenoidectomy effectiveness was followed up 6th and 12th month
Interventions: conventional curettage adenoidectomy versus endoscopic assisted adenoidectomy
Outcomes: operative time, blood loss, hospital stay, effective rate, complications
Name year: Feng Y. H. 2006
Methods: RCT (double blind)
Participants: 18 patients underwent endoscopic adenoidectomy and 16 underwent conventional curettage adenoidectomy, patients follow up from 6 to 12 months. All patients younger than 18 years old
Interventions: conventional curettage adenoidectomy versus endoscopic assisted adenoidectomy
Outcomes: operative time, blood loss, complications

### Outcome measures

In this study the main outcome measures were total operative time, blood loss and complications.

### Quality assessment

Because most of the relevant studies were randomized controlled trials, we chose the Jadad score for evaluation literature quality, which included randomization method, length of follow-up, and number of samples. A cumulative Jadad score ≥3 indicated high quality (Moher et al. 2000).

### Statistical analysis

The data extracted were pooled to obtain estimates of overall surgery effects using Review Manager for Windows version 5.3. The mean difference (MD) or standard mean difference (SMD) with its 95 % confidence interval (CI) was used and for dichotomous data, odds risk (OR) with a 95 % CI was applied. A value of p ≤ 0.05 indicated statistical significance. The statistical heterogeneity was assessed in our study using Cochrane’s Q test and I^2^ statistics. Clinical heterogeneity was evaluated by study interventions and the definition of outcome measures. A fixed-effects model also could be used when the heterogeneity was (p > 0.1 or p ≤ 0.1, but was I^2^ ≤ 50 %) and the significant heterogeneity (p ≤ 0.1, I^2^ ≥ 50 %), as described in detail by Wang et al. (2014).

## Results

### Search results and study characteristics

**Fig. 1:**
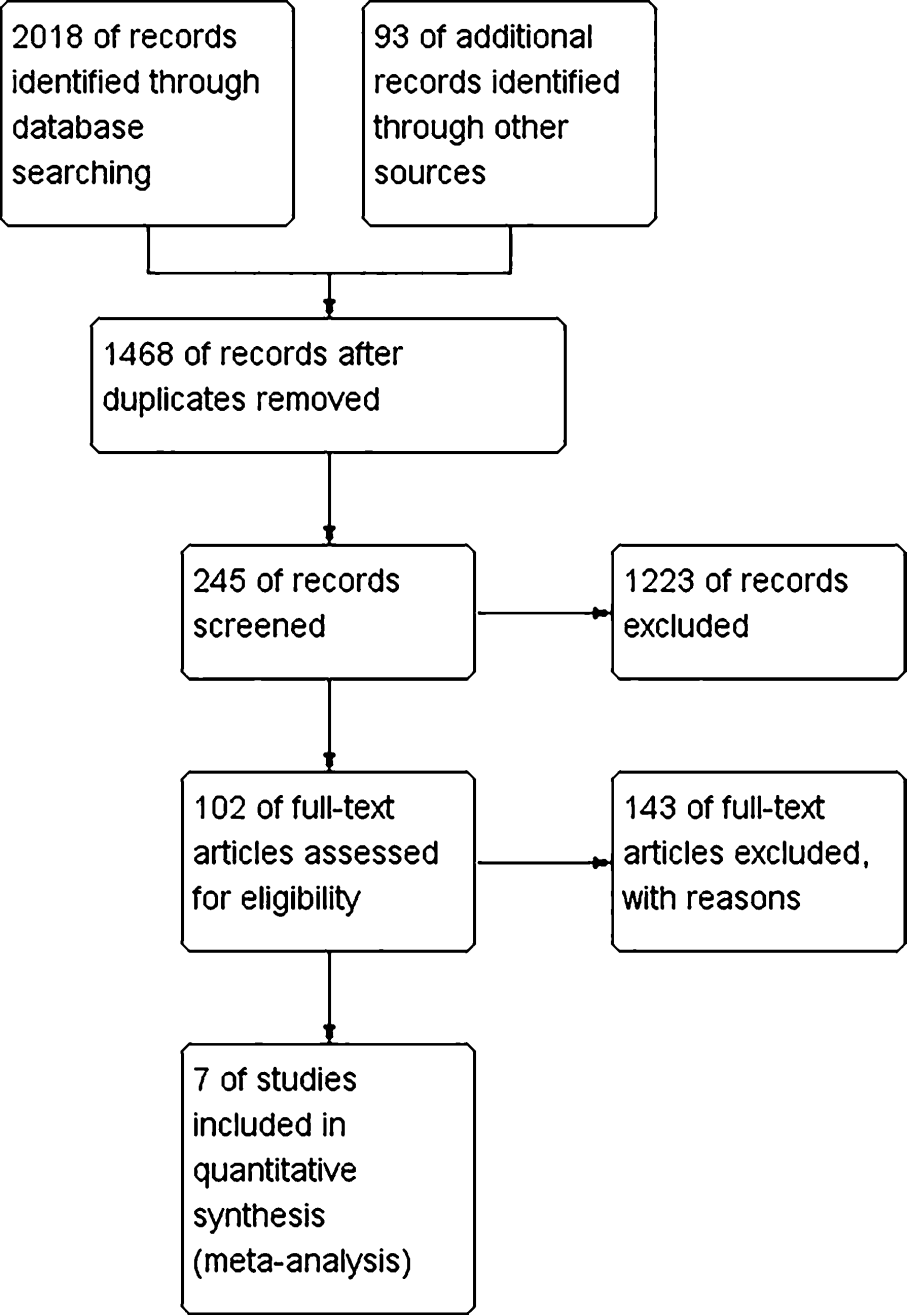
The flow diagram of included studies

With the key words, we found 2111 titles, of which 245 titles and abstracts were identified. Of these 245, 102 titles for which full texts were available were studied in detail. Unfortunately, most were retrospective non-randomized studies, case reports, or irrelevant review articles. The procedure for study selection is shown in Fig. 1. Ultimately, seven randomized controlled trials published from 2000 to 2013 matched the inclusion criteria (Songu et al. 2010; Al-Mazrou et al. 2009; Ozturk and Polat 2012; Stanislaw et al. 2000; Murray et al. 2002; Feng and Ying 2006; Zhang and Yang 2013). Five studies were published in English (Songu et al. 2010; Al-Mazrou et al. 2009; Ozturk and Polat 2012; Stanislaw et al. 2000; Murray et al. 2002) and two were published in Chinese (Feng and Ying 2006; Zhang and Yang 2013). A total of 582 patients in the studies underwent adenoidectomy, including 331 patients treated with endoscopic assisted adenoidectomy, and 251 patients with conventional curettage adenoidectomy. (For characteristics of adaptive trials refer to Table 1.)

### Quality assessment

**Fig. 2:**
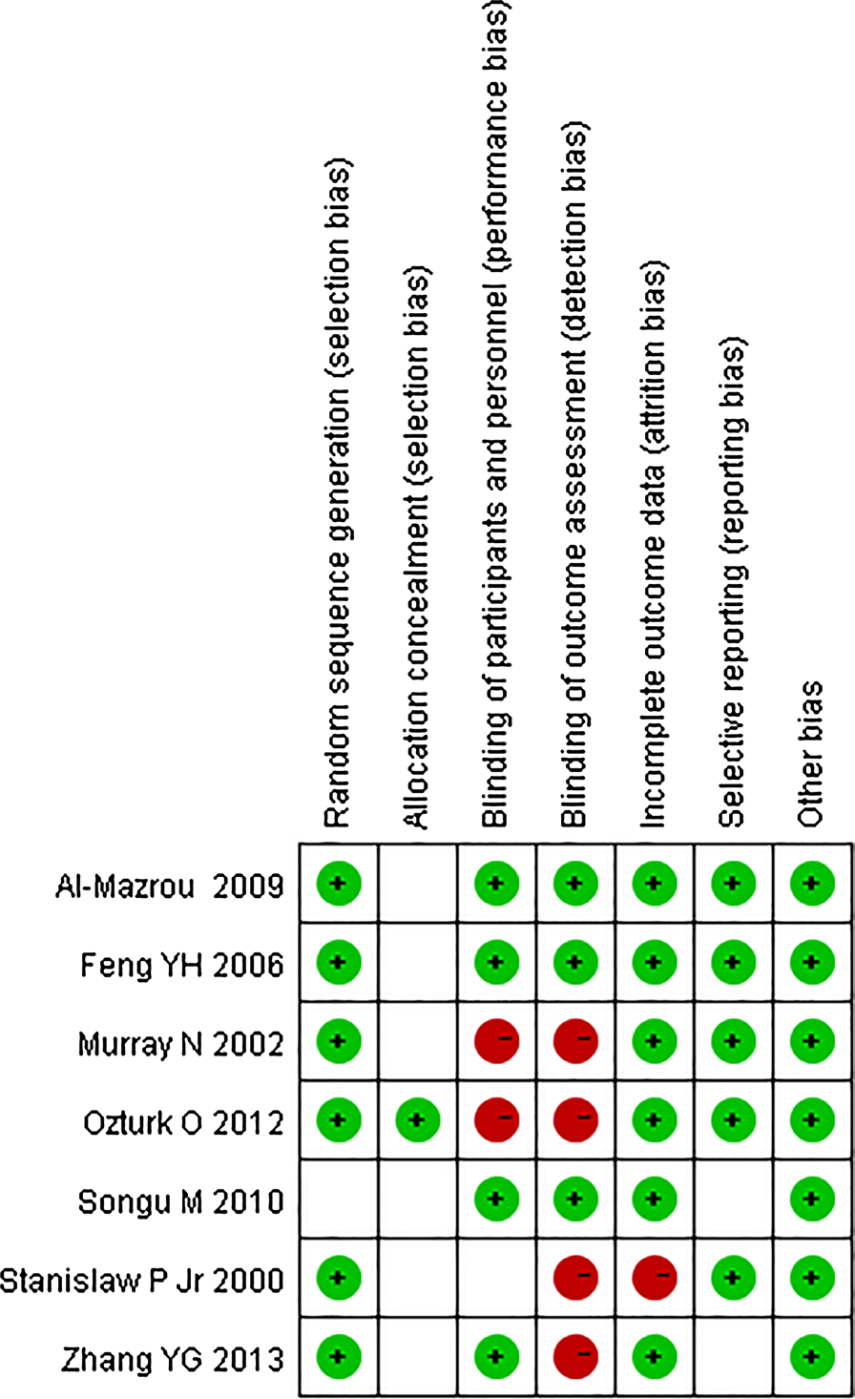
Risk of bias of included studies. Represents low risk represents high risk blank space of risk bias represents unclear risk

The study design, conduct and analysis were examined to assess quality. Both Songu et al. (2010) and Al-Mazrou et al. (2009) divided the children into groups using a computer-generated random numbers table and another study randomized subjects to treatment groups based on an odd or even medical number (Stanislaw et al. 2000). The remaining four studies did not clearly state the method used to randomize subjects to treatment groups. Only one study reported allocation to treatment with blinding using a worksheet (Ozturk and Polat 2012). A double-blind method was reported in three studies (Elnashar et al. 2014; Al-Mazrou et al. 2009; Feng and Ying 2006), and a single-blind method was mentioned in another study (Zhang and Yang 2013). The other three studies did not report blinding, so this was considered an undefined risk. The risks of bias for each of the seven studies are shown in Fig. 2.

### Outcomes and synthesis of results

#### Total operative time

**Fig. 3:**
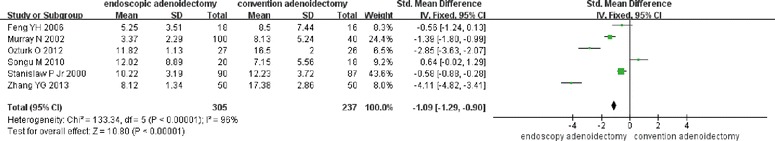
Forest plots of standard mean difference (SMD) and 95 % confidence interval (CI) for total operative time

Total operative time was reported in six studies. According to Fig. 3, in the meta-analysis showed that it was shorter in the endoscopic assisted adenoidectomy group than in the conventional curettage adenoidectomy group (SMD −1.09; 95 % CI −1.29 to −0.90; p < 0.00001) with statistical heterogeneity; (χ^2^ = 133.34, I^2^ = 96 %; p < 0.00001).

#### Blood loss

**Fig. 4:**
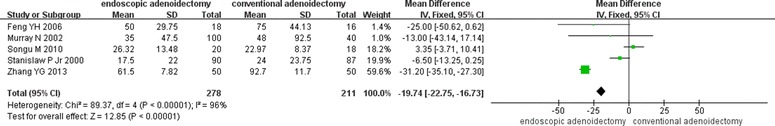
Forest plots of standard mean difference (SMD) and 95 % confidence interval (CI) for blood loss

As shown in Fig. 4, blood loss was compared in five studies the results of which showed that it was less in the endoscopic assisted adenoidectomy group than in the conventional curettage adenoidectomy group (MD −19.74; 95 % CI −22.75 to −16.73; p < 0.00001), with statistical heterogeneity (χ^2^ = 89.37, I^2^ = 96 %; p < 0.00001).

#### Complications

**Fig. 5:**
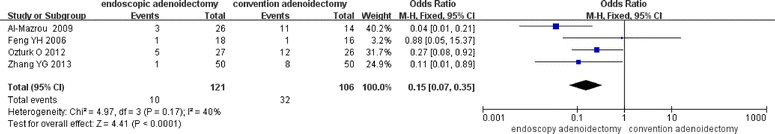
Forest plots of odds ratio (OR) and 95 % confidence interval (CI) for complications

According to Fig. 5, complications were reported in three of seven studies. The data showed that there were fewer complications in the endoscopic assisted adenoidectomy group than the conventional curettage adenoidectomy group (OR 0.15; 95 % CI 0.07–0.35, p < 0.0001), with statistical heterogeneity: (χ^2^ = 4.97, I^2^ = 40 %; p = 0.17).

## Discussion

Growing adenoid tissue is inclined to narrow the upper airway lumen to varying degrees (Papaioannou et al. 2013). Large adenoids and tonsils are diagnosed in children with mouth breathing, snoring, and sleep-disordered breathing (Niemi et al. 2015). The best way to solve the problem is adenoidectomy (Friedman et al. 2009) which must alleviate chronic nasal obstruction, mouth breathing, rhinosinusitis and eustachian tube dysfunction (Anand et al. 2014). In the clinic, the operative approaches include conventional curettage adenoidectomy and endoscopic assisted adenoidectomy. Here we report the first comprehensive meta-analysis comparing endoscopic assisted adenoidectomy and conventional curettage adenoidectomy in order to determine which method has greater benefits for children.

This meta-analysis included seven studies that all met the inclusion criteria (Songu et al. 2010; Al-Mazrou et al. 2009; Ozturk and Polat 2012; Stanislaw et al. 2000; Murray et al. 2002; Feng and Ying 2006; Zhang and Yang 2013). Five studies stated the total operative time, and the result of meta-analysis showed that endoscopic assisted adenoidectomy was better than conventional curettage adenoidectomy in this regard. The related shorter operative time could be explained by the endoscopic adenoidectomy is an operation to remove pathological tissues which clearly block the choana, and the shaver can reach them directly by using 0°, 30° endoscopy (Somani et al. 2010). Although the actual procedure time is not concerned, the total operative time the children spent is possible more important. This is specifically important in children with upper airway obstruction where the time taken to induce adequate anaesthesia and the time taken for the patient to emerge from anaesthesia was often long and unpredictable (Songu et al. 2010).

Five studies selected reported blood loss, and the meta-analysis results demonstrated that the conventional curettage adenoidectomy caused greater blood loss than endoscopic assisted adenoidectomy. This is mainly related to direct visualization, treatment of the source of bleeding, the effect of a microdebrider depended on suction efficacy and hemostasis is noticeably shortened (Vokurka 2003). This reduction of blood loss is highly suitable for children because it reduces the risk of hemorrhage (Al-Mazrou et al. 2009).

Four studies reported complications associated with endoscopic assisted adenoidectomy and conventional curettage adenoidectomy. Our meta-analysis showed that the former was superior to the latter. Concerning the intraoperative view the conventional technique gives worse visualization of the operative field which is liable to miss tissues, leading to relapse with inflammation (Regmi et al. 2011). In the clinic, conventional technique increases the risk of damaging the eustachain tube openings in the region of the rhinopharynx (Viorel 2011) and leads to mild hearing loss (Capaccio et al. 2016). Furthermore, the adenoids may only be reduced, not completely removed. If the adenoids are not completely removed, they may continue to be re-grow and cause airway obstruction (Al-Mazrou et al. 2009). By contrast, endoscopic assisted adenoidectomy removes the tissues cleanly and solves the nasopharyngeal obstruction completely.

The results of our meta-analysis suggest that the endoscopic assisted adenoidectomy is better than conventional curettage adenoidectomy in terns of total operative time, blood loss, and complications.

Our meta-analysis focused on objective outcome measurements to define surgical effectiveness. Nevertheless, dependence on subjective measurements may overestimate effectiveness. A few limitations of this meta-analysis should be considered. First, there only seven studies were included; limitations of meta-analysis based on the number of eligible studies are well known, including confounding factors and selection bias, so the results must be viewed with caution. Second, there were some differences among studies in the basic definition of effectiveness. Therefore, effectiveness differs among the studies presented, contributing to heterogeneity.

## Conclusion

According to our meta-analysis, endoscopic assisted adenoidectomy has advantages over conventional curettage adenoidectomy with regard to total operative time, blood loss and complications. This result finding may be useful to otolaryngologists when choosing surgery for adenoid hypertrophy. However, current evidence is incomplete, and we hope that more high quality clinical studies will be published to enhance meta-analysis outcomes and provide a better guide for surgeons.
